# Blended versus face-to-face cognitive behavioural therapy for severe fatigue in patients with multiple sclerosis: A non-inferiority RCT

**DOI:** 10.1177/13524585231185462

**Published:** 2023-07-25

**Authors:** Marieke de Gier, Heleen Beckerman, Jos Twisk, Hans Knoop, Vincent de Groot

**Affiliations:** Department of Medical Psychology, Amsterdam UMC Location Vrije Universiteit Amsterdam, Amsterdam, The Netherlands; MS Center Amsterdam, Amsterdam Neuroscience Research Institute, Amsterdam, The Netherlands; Amsterdam Public Health Research Institute, Amsterdam, The Netherlands; Department of Rehabilitation Medicine, Amsterdam UMC Location Vrije Universiteit Amsterdam, Amsterdam, The Netherlands; MS Center Amsterdam, Amsterdam Public Health Research Institute, Amsterdam, The Netherlands; Department of Epidemiology and Data Science, Amsterdam UMC Location Vrije Universiteit Amsterdam, Amsterdam, The Netherlands; Department of Medical Psychology, Amsterdam UMC Location Vrije Universiteit Amsterdam, Amsterdam, The Netherlands; Department of Medical Psychology, Amsterdam UMC Location University of Amsterdam, Amsterdam, The Netherlands; Amsterdam Public Health Research Institute, Amsterdam, The Netherlands; Department of Rehabilitation Medicine, Amsterdam UMC Location Vrije Universiteit Amsterdam, Amsterdam, The Netherlands; MS Center Amsterdam, Amsterdam Public Health Research Institute, Amsterdam, The Netherlands

**Keywords:** MS-related fatigue, cognitive behavioural therapy, e-health, symptom management, rehabilitation medicine

## Abstract

**Background:**

Cognitive behavioural therapy (CBT) reduces multiple sclerosis (MS)-related fatigue. Implementation of face-to-face CBT is hindered by limited treatment capacity and traveling distances to treatment locations.

**Objective:**

Evaluate whether blended CBT (online treatment modules supported with guidance by a therapist) is non-inferior to face-to-face CBT in reducing fatigue severity in severely fatigued patients with MS.

**Method:**

A non-inferiority multicentre randomized clinical trial, in which 166 patients with MS were allocated to either face-to-face or blended CBT. Primary outcome was fatigue severity assessed with the Checklist Individual Strength fatigue subscale directly post-treatment (week 20). Mixed model analysis was used by a statistician blinded for allocation to determine between-group differences post-treatment. The upper limit of the 95% confidence interval (CI) was compared to a pre-specified non-inferiority margin of 5.32.

**Results:**

Blended CBT (*N* = 82) was non-inferior to face-to-face CBT (*N* = 84) *(B* = 1.70, 95% CI: −1.51 to 4.90). Blended CBT significantly reduced therapist time (*B* = −187.1 minutes, 95% CI: 141.0–233.3). Post hoc analysis showed more improvement (*B* = −5.35, 95% CI: −9.22 to −1.48) when patients received their preferred treatment. No harm related to treatment was reported.

**Discussion:**

Blended CBT is an efficient alternative to face-to-face CBT. Offering the preferred CBT format may optimize treatment outcome.

## Introduction

Severe fatigue is a highly prevalent and burdensome symptom in people with multiple sclerosis (MS).^1–3^ The aetiology of MS-related fatigue is likely to be multifactorial. The cognitive behavioural model of MS-related fatigue assumes that disease-specific factors such as (neuro)inflammation trigger fatigue, but cognitive behavioural variables can perpetuate fatigue.^4^

Cognitive behavioural therapy (CBT) for MS-related fatigue aims to decrease fatigue by changing fatigue-related behaviours and beliefs.^5,6^ Recent meta-analyses and systematic reviews have shown CBT to be an effective treatment of MS-related fatigue.^7–11^ However, face-to-face CBT is an intensive treatment for both patients and therapists, while therapist capacity is often limited.

E-health interventions decrease travelling for patients to the outpatient clinic and allow patients to follow CBT in their own time and pace. This may be attractive considering the nature of the condition. Blended CBT, consisting of online treatment modules and face-to-face consultations with a psychologist, has been proven effective in TREating FAtigue in MS (TREFAMS) and other chronic conditions.^12–15^ Guidance from a therapist appeared to enhance treatment outcome as well as adherence in patients with MS, compared to online CBT without guidance.^15^

Thus far, effectiveness of blended CBT in reducing fatigue has not directly been compared to face-to-face CBT. The aim of this study was to determine whether blended CBT is non-inferior with respect to its effect on fatigue severity compared with face-to-face CBT in severely fatigued patients with MS. Furthermore, it was investigated whether blended CBT required less therapist time compared to face-to-face CBT and whether treatment is more effective when applied in the format congruent with the preference of the patient.

## Methods

### Trial design

We conducted an observer-blinded non-inferiority multicentre randomized clinical trial (RCT), with patients being allocated to face-to-face or blended CBT. In all treatment sites, therapists delivered both CBT formats.^16^

The non-inferiority margin was based on the results of the TREFAMS-CBT study that tested the effectiveness of CBT compared to MS nurse consultations. The between-group difference for fatigue was 6.67 points (95% confidence interval (CI): 2.70–10.68) on the Checklist Individual Strength (CIS) fatigue severity subscale.^17^ The non-inferiority margin was calculated as the mean effect 6.67 – 50% of the lower limit (i.e. 2.7) of the 95% CI of the TREFAMS-CBT effect, that is, 6.67–1.35 = 5.32.^18^

The study was approved by the Medical Ethical Review Committee, Amsterdam University Medical Centres (registration 2017.538, NL62622.029.17), and by the local ethical committees of participating hospitals and rehabilitation centres. The study was registered in the Dutch Trial Register (NTR6966; registered 18 January 2018 https://www.trialregister.nl/trial/6782). The study protocol has been published.^19^

### Participants

Participating MS patients had to meet the same inclusion criteria as the TREFAMS-CBT study:^18,,19,21,22^ (a) definitive diagnosis of MS, confirmed by a neurologist; (b) severely fatigued, CIS fatigue ⩾ 35; (c) aged between 18 and 70 years; (d) Expanded Disability Status Scale (EDSS) ⩽ 6); (e) no signs of an exacerbation and no corticosteroid treatment in the past 3 months; and (f) no clinical indication of current infections, anaemia or thyroid dysfunction. Exclusion criteria were (a) depressive disorder, assessed with the Beck Depression Inventory-Primary Care version (BDI-PC)^23^ and Mini-International Neuropsychiatric Interview (M.I.N.I.).^24^ In case of a BDI score > 3, the depression module of the M.I.N.I. was administered; (b) primary sleep disorders; (c) severe co-morbidity (Cumulative Illness Rating Scale^25^ ⩾ 3); (d) pregnancy or having given birth in the past 3 months; (e) pharmacological treatment for fatigue that was started in the past 3 months; (f) non-pharmacological therapies for fatigue in the last 3 months; and (g) having received CBT for fatigue.

MS patients were recruited from participating hospitals and rehabilitation centres and by advertising on social media of MS patient associations. Patients could be referred by their rehabilitation physician or neurologist (*N* = 145). Self-referred patients (*N* = 21) were first examined by a rehabilitation physician to exclude co-morbidity that could explain the presence of fatigue. Patients were informed about the study by a research assistant, and after giving written consent, completed the CIS fatigue severity subscale^26^ and the BDI-PC^23^ online to determine whether they were severely fatigued (CIS fatigue ⩾ 35) and had no clinically relevant depressive symptoms (BDI ⩽ 3). All eligibility criteria were determined by the research psychologist during a semi-structured interview by telephone, eventually consulting a rehabilitation physician.

### Interventions

CBT for MS-related fatigue is directed at decreasing fatigue by changing fatigue-maintaining cognitions and behaviours. The CBT was patient-tailored by determining which of the 10 treatment modules applied to the individual patient on the basis of scores on questionnaires and the clinical judgement of the therapist during the first session. The content of the treatment modules and cut-off scores on questionnaires used for patient-tailoring CBT are described in Supplement 1.

### Face-to-face CBT

Face-to-face CBT consisted of 12 individual, face-to-face, 45-minute therapy sessions in 20 weeks according to the protocol previously used in the TREFAMS-CBT study^18^ (Supplement 1).

### Blended CBT

‘MS Fit’ was developed based on the face-to-face CBT protocol.^18^ MS Fit consisted of two face-to-face contacts, respectively, at the beginning and at the end of the 20-week treatment period. During the 20 weeks, patients received tailored information and treatment assignments in the online MS Fit portal, supported by three to four video consultations and optional email contact with their therapist (see Supplement 1).

For both intervention groups, therapists registered which modules were indicated and addressed during treatment. In addition, they registered the form (face-to-face, video, phone, email) and duration of each contact. Log data of the online platform provided information about which treatment modules were opened and completed by the patient.

All therapists were certified psychologists who received a 4-day training in the treatment protocols and received bi-weekly supervision by an experienced clinical psychologist (HK or MdG) to ensure treatment integrity.

### Protocol deviations

Halfway of RCT, the COVID-19 pandemic occurred influencing our original study protocol^19^ since, in accordance with the local measures of the treatment sites, some face-to-face sessions were substituted by video consultations.

### Adherence

Treatment adherence for the face-to-face CBT was defined as attending at least three treatment sessions, including a session at the end of the 20-week period. For the blended CBT, adherence was defined as attending the first and last treatment sessions, and opening at least the MS Fit modules ‘sleep and rest’ and ‘physical activity regulation’ as an indication that patients actively started MS Fit. Adherence also included not receiving other treatments for fatigue and not shifting the focus of treatment to other occurring co-morbidities such as depression. Patients who dropped out during the treatment period were requested to complete the questionnaires at week 20.

### Outcomes

All outcome measures were assessed online at baseline before randomization and at the end of the 20-week treatment period. Treatment preference was assessed with one question in the online assessment at baseline.

The primary outcome was fatigue severity assessed with the CIS fatigue severity subscale.^18^ The CIS is a fatigue questionnaire, consisting of four subscales assessing fatigue severity (eight items), reduction in motivation (four items), reduction in physical activity (three items) and concentration problems (five items). The items are answered on a 7-point Likert-type scale and the total score of the fatigue severity subscale varies between 8 and 56 points. A score of 35 or higher is indicative for severe fatigue.^17^ The CIS is a reliable and valid instrument.^26^ Clinically significant improvement was defined as either a score of < 35 on the CIS subscale fatigue severity, or an improvement of ⩾ 8 points on this subscale.^18^

Secondary outcome measures were other fatigue measures (Fatigue Severity Scale and PROMIS-Fatigue Short Form 8a^27–29^), limitations in daily functioning and quality of life (Sickness Impact Profile, Work and Social Adjustment Scale and SF36^30–33^). See Supplement 2 for a description of the psychometric qualities of these questionnaires.

The BDI-PC was used to assess depressive symptoms. The BDI-PC consists of seven items, scored on a 4-point scale ranging from 0 to 3, adding to a total score with a maximum of 21. A total score > 3 is considered indicative for depression.^23^

### Sample size

Sample size calculation was based on the point estimate of the treatment effect of CBT in the previous study, that is, 6.67, and the SD 11.6 of the CIS fatigue from the TREFAMS-CBT study group.^18^ In order to be 80% sure that the upper limit of a one-sided 95% CI would be below the non-inferiority margin of 5.32 points on the CIS fatigue severity subscale, 150 participants were required. Adjusting for a drop-out of 10%, 166 MS patients needed to be included.

### Randomization

Randomization with concealed treatment allocation was carried out by a project member not involved in the enrolment or assessments, using a web-based randomization facility within CASTOR EDC. The randomization scheme was computer-generated with stratification for treatment centre and using random block sizes (range 2–6). The outcome of the randomization was reported to the therapist by the research psychologist.

### Blinding

Patients and therapists could not be blinded for the intervention. The research assistant responsible for the assessments and the statistician conducting the statistical analyses were blinded for treatment allocation.

### Serious adverse events

Serious adverse events (SAEs) were reported to the researcher by the therapist or participant. SAEs were reported for review to the Medical Ethics Committee.

### Statistical analyses

#### Primary analysis non-inferiority

Statistical analyses were pre-specified and conducted according to the intention-to-treat (ITT) principle, including all 166 randomized participants, and on the per-protocol sample, including patients who met criteria for adherence.^16,22^ Multilevel analyses were conducted with three levels as (a) patient, (b) therapist and (c) treatment centre, with CIS fatigue post-treatment as the dependent variable. Treatment group was a fixed factor and CIS fatigue score at baseline was added as covariate. The multilevel analysis was repeated with clinically significantly improved patients (yes/no) as outcome. Post hoc, the primary analysis was repeated excluding participants in the face-to-face condition with more than two video consultations due to COVID-19 regulations.

#### Secondary outcomes

The multilevel analyses were repeated for the secondary outcome measures. The secondary analyses were not part of the primary, non-inferiority analysis. Therefore, between-group differences were tested using a two-sided 95% CI.

Differences in therapist time between both interventions were analysed in patients who at least attended the first therapy session.

#### Treatment preference

In addition to the published study protocol,^19^ the primary analysis was repeated with treatment preference (congruent/incongruent with allocated treatment/no preference) as fixed factor.

## Results

### Patients

**Figure 1.**
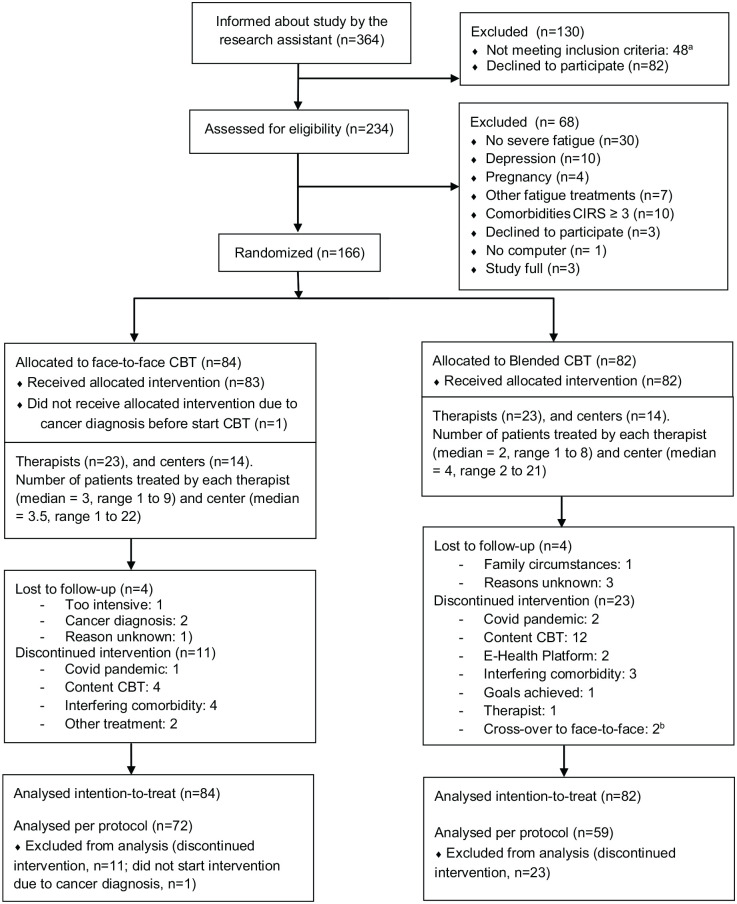
Consort flow diagram. ^a^Indicated by patients in response to the information provided by the research assistant; no severe fatigue (*n* = 10), MS-exacerbation (*n* = 1), age > 70 (*n* = 1), other fatigue treatments (*n* = 12), comorbidities (*n* = 14), not ambulatory (*n* = 7), no diagnosis MS (*n* = 3). ^b^e-health platform (*n* = 1), comorbid depression and cognitive problems (*n* = 1).

Between April 2018 and December 2021, 364 MS patients were actively informed by the research team about the study, of whom 234 signed informed consent and were screened for eligibility. In total, 166 MS patients (75.9% female, mean age 45.5, median disease duration of 9.1 years) were randomized (see Table 1). Twenty-six therapists from 14 participating centres provided both therapies. In both treatment arms, three patients were lost to follow-up. In the face-to-face CBT group 73 patients (87%) adhered to the allocated treatment, compared to 59 patients (71%) in the blended CBT group. Reasons for non-adherence are presented in Figure 1 (Flow diagram).

**Table 1.** Baseline characteristics of the 166 trial participants with MS-related fatigue.

	Face-to-face CBT (***N*** = 84)	Blended CBT (***N*** = 82)
Age in years (mean, SD)	45.4 (10.7)	45.7 (9.9)
Gender		
Female	64 (76.2%)	62 (75.6%)
Level of education		
Low	6 (7.1%)	7 (8.5%)
Medium	33 (39.3%)	36 (43.9%)
High	45 (53.6%)	39 (47.6%)
Civil status		
Living alone	17 (20.2%)	12 (14.6%)
Living with partner	59 (70.2%)	60 (73.2%)
Employment status		
Full time	8 (9.5%)	7 (8.5%)
Part time	24 (28.6%)	30 (36.6%)
On disability pension (partly)	19 (22.6%)	14 (17.1%)
Disability pension (full)	30 (35.7%)	31 (37.8%)
Unemployed	12 (14.3%)	20 (24.4%)
Retired	4 (4.8%)	0
Student	2 (2.4%)	0
Time since diagnosis in years (median, min–**max)**	7.0 (0–41)	7.0 (0–41)
EDSS (median, min–max)	3.5 (1–6)	3.5 (0–6)
Type of MS		
RRMS	67 (79.8%)	66 (80.5%)
PPMS	7 (8.3%)	6 (7.3%)
SPMS	8 (9.5%)	9 (11.0%)
Unknown	2 (2.4%)	1 (1.2%)
CIS fatigue	45.6 (6.0)	45.2 (6.3)
SIP8 total	1599.8 (607.5)	1724.7 (641.7)
SF36 physical functioning	61.85 (23.0)	59.82 (21.7)
WSAS	20.8 (7.5)	20.3 (7.8)
BDI	3.1 (2.2)	3.5 (2.5)
**Treatment preference**		
Face-to-face	35 (41.7%)	36 (43.9%)
Blended	25 (29.8)	17 (20.7%)
No preference	24 (28.6%)	29 (35.3%)

MS: multiple sclerosis; CBT: cognitive behavioural therapy; EDSS: expanded disability status scale; RRMS: relapsing remitting multiple sclerosis; PPMS: primary progressive multiple sclerosis; SPMS: secondary progressive multiple sclerosis; CIS: checklist individual strength; SIP: sickness impact profile; SF36: short form 36; WSAS: work and social adjustment scale; BDI: Beck Depression Inventory.

### Non-inferiority

**Figure 2.**
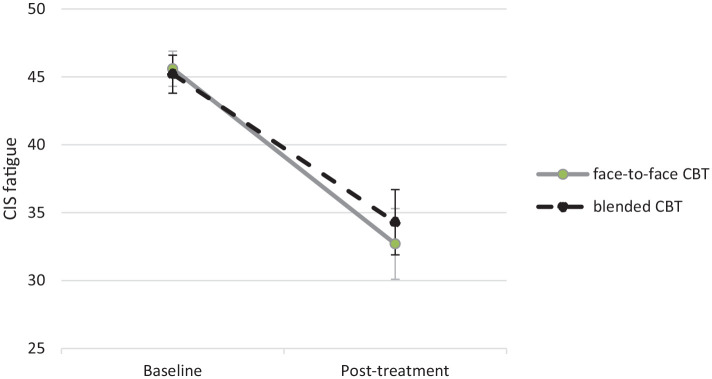
Fatigue severity pre- and post-treatment in both treatment conditions (mean and 95% CI).

Change scores and differences between treatment groups on all outcomes are reported in Table 2. Figure 2 shows the decrease in CIS fatigue scores of the treatment conditions. In both the ITT analysis and the per-protocol analysis, the upper bound of the 95% CI of the difference between both treatments was below the non-inferiority margin of 5.32 (*B* = 1.70, 95% CI: −1.51 to 4.90 and *B* = 1.46, 95% CI: −2.07 to 4.98, respectively) slightly favouring face-to-face CBT. Treatment effects did not depend on treatment centre or therapist.

**Table 2.** Mean change scores of the primary and secondary outcome measures and the differences between treatment groups corrected for baseline.

	Face-to-face CBT			Blended CBT			
	T0 (*n* = 84)	T20 (*n* = 80)	Within-group changes	T0 (*n* = 82)	T20 (*n* = 78)	Within-group changes	Between-group effect corrected for baseline
	Mean (SD)	Mean (SD)	Mean (95% CI)	Mean (SD)	Mean (SD)	Mean (95% CI)	*B* (95% CI)
CIS fatigue	45.6 (6.0)	32.7 (11.8)	−12.6 (−15.2 to −10.0)	45.2 (6.3)	34.3 (10.8)	−11.0 (−13.2 to −8.8)	1.70 (−1.5 to 4.9)
FSS	5.3 (0.9)	4.2 (1.2)	−1.1 (−1.4 to 0.8)	5.4 (0.9)	4.5 (1.0)	−0.9 (−1.1 to −0.7)	0.28 (−0.1 to 0.6)
PROMIS-Fatigue	29.7 (4.1)	21.3 (6.8)	−8.2 (−9.9 to −6.6)	29.8 (4.9)	22.7 (6.4)	−7.2 (−8.7 to −5.6)	1.33 (−0.7 to 3.3)
SF36 physical function	61.9 (23.0)	70.2 (23.3)	8.3 (5.0 to 11.7)	59.8 (21.7)	67.4 (21.8)	7.6 (4.8 to 10.4)	−1.04 (−5.2 to 3.2)
SF36 role physical	21.7 (30.0)	55.3 (40.3)	33.1 (23.8 to 42.4)	18.3 (28.1)	51.0 (38.5)	33.2 (24.3 to 42.1)	−2.16 (−13.8 to 9.5)
SF36 role emotion	65.9 (39.4)	82.1 (31.8)	14.6 (3.5 to 25.7)	66.3 (42.1)	79.0 (36.2)	10.0 (−0.7 to 20.8)	−3.42 (−13.8 to 6.9)
SF36 vitality	37.9 (13.9)	55.8 (18.5)	17.3 (13.3 to 21.2)	34.3 (13.4)	53.2 (18.0)	19.3 (15.4 to 23.2)	−0.06 (−5.2 to 5.0)
SF36 emotional wellbeing	69.9 (17.6)	76.1 (15.3)	5.9 (2.8 to 8.9)	69.7 (14.3)	77.0 (15.1)	6.3 (3.4 to 9.2)	0.64 (−2.8 to 4.2)
SF36 social function	55.2 (21.1)	69.5 (23.4)	13.3 (7.8 to 18.7)	55.8 (23.3)	71.4 (20.1)	16.1 (10.9 to 21.3)	2.20 (−4.1 to 8.5)
SF36 pain	69.8 (24.0)	73.8 (20.0)	2.7 (−1.0 to 6.4)	66.6 (24.7)	71.7 (20.4)	4.9 (0.2 to 9.6)	0.30 (−4.6 to 5.1)
SF36 general health	43.1 (18.5)	51.1 (18.7)	7.4 (3.7 to 11.1)	41.9 (18.0)	51.2 (21.0)	9.0 (5.9 to 12.2)	1.22 (−4.6 to 5.1)
SIP8 total	1599.8 (607.5)	1179.3 (764.0)	−385.5 (−520.1 to −251.0)	1724.7 (641.7)	1181.5 (711.7)	−500.4 (−633.1 to −367.7)	−84.98 (−267.1 to 97.2)
WSAS total	20.8 (7.5)	16.3 (7.9)	−4.0 (−5.4 to −2.6)	20.3 (7.8)	16.7 (7.9)	−3.8 (−5.3 to −2.2)	0.29 (−1.6 to 2.2)
BDI	3.1 (2.2)	1.9 (2.1)	−1.2 (−1.6 to −0.7)	3.5 (2.5)	1.9 (2.0)	−1.4 (−1.8 to −1.0)	−0.11 (−0.6 to 0.4)
% clinically relevant change^a^		63.8%			66.6%		0.13 (−0.56 to 0. 83)
Time treatment (minutes)		574.1 (146.4)			394.65 (155.8)		−187.1 (−141.0 to −233.3)

CBT: cognitive behavioural therapy; CI: confidence interval; CIS: checklist individual strength; FSS: fatigue severity scale; SF36: short form 36; SIP: sickness impact profile; WSAS: work and social adjustment scale; BDI: Beck Depression Inventory.

a Clinically relevant change = either CIS fatigue post-treatment < 35, or a minimal change of 8 points on the CIS fatigue.

In the face-to-face group, 63.8% of the patients showed a clinically relevant improvement of fatigue severity, and 66.6% in the blended condition (63.6% in the TREFAMS-CBT study^18^). Blended CBT saved 3 hours of therapist time compared to face-to-face CBT (*B* = −187.1 minutes, 95% CI: −141.0 to −233.3 minutes).

Post hoc ITT analysis, excluding (*n* = 27) participants in the face-to-face condition who due to COVID regulations received three or more video consultations, showed similar between-group effects (*B* = 1.05, 95% CI: −2.24 to 4.33).

### Secondary outcomes

The differences between face-to-face CBT and blended CBT were small and non-significant on all secondary outcomes (see Table 2). Effect sizes, expressed as Cohen’s *d*, ranged from 0.00 to 0.27. The significant within-group improvements on FSS, PROMIS-fatigue and SF36 vitality do support the effect of both interventions on fatigue. Applying the cut-off score of 5 on the FSS^34^ showed that 71% of the patients in the face-to-face condition and 63% in the blended condition were no longer severely fatigued after treatment.

### Treatment preference

**Figure 3.**
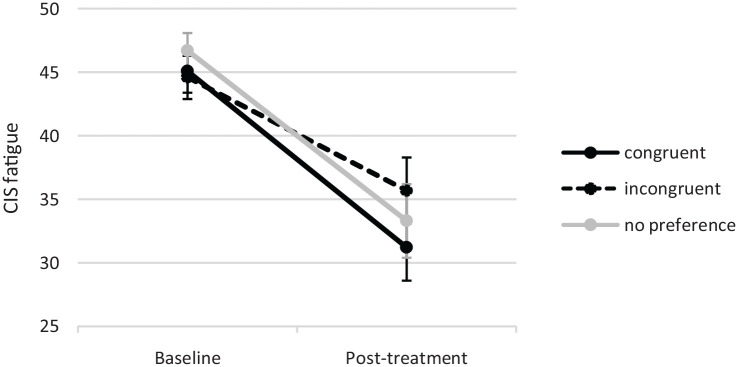
Effect of congruence of treatment preference with treatment allocation on outcome (mean and 95% CI).

Patients receiving their preferred treatment profited significantly more from CBT compared to patients who were allocated to the treatment incongruent with their preference (*B* = −5.35, 95% CI: −9.22 to −1.48). Despite this difference, the latter group still showed a clinically relevant treatment response, (see Table 3 and Figure 3). Post hoc analysis showed that treatment adherence was significantly larger in patients who received the preferred treatment (90.4%) compared to the non-preferred treatment (72.6%) or no preference (76.9%; Chi-square = 17.5, *p* < 0.001).

**Table 3.** Mixed linear model with fatigue severity post-treatment as dependent variable.

	Effect (*B*)	95% CI	*p*
(Constant)	3.81	−8.72 to 16.35	0.551
CIS_fatigue at baseline	0.60	0.34 to 0.87	< 0.001
Congruence treatment with preference			
Congruent with preference (ref)	–	–	–
Incongruent with preference	5.35	1.48 to 9.22	0.007
No preference	1.47	−2.49 to 5.43	0.466

CI: confidence interval; CIS: checklist individual strength.

### Serious adverse events

In the blended CBT group, one patient was hospitalized due to an MS exacerbation. In the face-to-face group, five SAEs were reported: diagnosis cancer (*n* = 3), pulmonary embolism (*n* = 1), hospitalized due to MS exacerbation (*n* = 1). All SAEs were reported to the medical ethics committee and judged to be unrelated to the interventions.

## Discussion

This is the first study testing non-inferiority of blended CBT to face-to-face CBT in treating MS-related fatigue. Both the ITT and per-protocol analysis show blended CBT (MS Fit) to be non-inferior to face-to-face CBT according to the predefined non-inferiority margin. Blended CBT was more efficient, requiring 3 hours less therapist time per patient to deliver. In both CBT formats, the majority of patients reported a clinically relevant change in fatigue. Results further indicate that patients profit more from CBT when they received the CBT format they preferred.

Patients in the face-to-face condition were more adherent to the treatment compared to patients in the blended condition. Adherence was larger when patients received the preferred treatment format. Content of CBT was mentioned more often as reason for non-adherence in the blended condition compared to the face-to-face condition. Since the content of the online modules is written more general, as opposed to a personalized rationale by a therapist, this may appeal stronger to the ability of patients to translate the rationale to their personal situation. Despite the larger treatment drop-out, the proportion of clinically relevant responders in the blended CBT was similar to the face-to-face condition. Face-to-face CBT apparently enhances adherence, with no discernible larger effect on fatigue compared to blended CBT. Studying which patient characteristics predict adherence and effectiveness of both face-to-face and blended CBT would be of great clinical relevance and helpful in personalizing treatment.

One important assumption in a non-inferiority RCT is that the effectiveness of the standard treatment (i.e. face-to-face CBT) is preserved in the non-inferiority RCT. Therefore, the same treatment protocol, and the same enrolment criteria should be used.^20,22^ Even though these criteria were met in our study, it should be taken into consideration that the duration of the treatment was extended to 20 weeks instead of 16 weeks. On the basis of clinical experiences with the original treatment protocol, it was concluded that patients often need more time to reach the goals they set in the CBT, and may have been more successful in this regard with four more weeks.

### Strengths and limitations

Conducting this multicentre RCT implicated training and supervising 26 psychologists in 14 hospitals and rehabilitation centres, which in itself led to simultaneous implementation of CBT for MS-related fatigue in a large part of the Netherlands and in an MS centre in Belgium. Even though treatment centres may vary in patient populations, and therapists may differ in their experience in providing (blended) CBT, treatment effects did not depend on treatment centre or therapist. Implementation of both forms of CBT seems to be possible irrespective of these factors.

We have no data on cost-effectiveness of blended CBT compared to face-to-face CBT. Although blended CBT saves therapist time, there are costs of the access to and maintenance of the e-health platform. Costs may depend on the scale on which MS Fit can be applied within a treatment centre and whether the platform is used for other e-health programmes as well.

A debatable consideration of this study is the choice of the non-inferiority margin, which may have been too liberal.^35^ In the published study protocol two methods of determining the non-inferiority margin were unintendedly mixed. The fixed-margin method, recommended by the FDA,^22,36^ determines the margin as 50% of the lower limit of the 95% CI of the effect found in the previous study, that is, 1.35. The margin in the point-estimate method is based on the point-estimate itself (i.e. 6.67), and the fraction of the point estimate to be preserved.^36^ The size of the acceptable loss is debatable, and the smaller the chosen margin, the larger the sample size needed. Applying a non-inferiority margin of 1.35 using the fixed margin method, would have required a sample size of 876 participants, which is not realistic in a Dutch research setting. This illustrates the importance of statistical and clinical reasoning in determining the margin of non-inferiority and the challenges of conducting non-inferiority trials in behavioural and rehabilitation medicine as it requires large sample sizes.

This study included ambulatory patients (EDSS ⩽ 6) without severe somatic comorbidity and/or depression. Future research needs to confirm effectiveness of CBT for fatigue in wheelchair-bound patients.

Results of this non-inferiority study are limited to fatigue severity directly post-treatment. However, patients were randomized again at the end of treatment, to either receiving a blended booster programme, or no booster programme, in order to test whether a booster is superior to improve long-term effects of CBT for fatigue. Results of this study will be presented separately.

## Conclusion

Blended CBT can be considered an effective alternative to face-to-face CBT, saving 3 hours of therapist time. Offering CBT in the preferred format might further optimize treatment outcome. When patients have no treatment preference, efficiency may be an argument to start blended CBT.

## Supplemental Material

sj-docx-1-msj-10.1177_13524585231185462 – Supplemental material for Blended versus face-to-face cognitive behavioural therapy for severe fatigue in patients with multiple sclerosis: A non-inferiority RCT Click here for additional data file. Supplemental material, sj-docx-1-msj-10.1177_13524585231185462 for Blended versus face-to-face cognitive behavioural therapy for severe fatigue in patients with multiple sclerosis: A non-inferiority RCT by Marieke de Gier, Heleen Beckerman, Jos Twisk, Hans Knoop and Vincent de Groot in Multiple Sclerosis Journal

sj-docx-2-msj-10.1177_13524585231185462 – Supplemental material for Blended versus face-to-face cognitive behavioural therapy for severe fatigue in patients with multiple sclerosis: A non-inferiority RCT Click here for additional data file. Supplemental material, sj-docx-2-msj-10.1177_13524585231185462 for Blended versus face-to-face cognitive behavioural therapy for severe fatigue in patients with multiple sclerosis: A non-inferiority RCT by Marieke de Gier, Heleen Beckerman, Jos Twisk, Hans Knoop and Vincent de Groot in Multiple Sclerosis Journal
