# Supplementary material for: Blended versus face-to-face cognitive behavioural therapy for severe fatigue in patients with multiple sclerosis: A non-inferiority RCT
Source: Mult Scler. 2023 Jul 25;29(10):1316–26. doi: 10.1177/13524585231185462 (PMC10503237; doi:10.1177/13524585231185462)
Supplement: sj-docx-1-msj-10.1177_13524585231185462 – Supplemental material for Blended versus face-to-face cognitive behavioural therapy for severe fatigue in patients with multiple sclerosis: A non-inferiority RCT [file sj-docx-1-msj-10.1177_13524585231185462.docx]

**Supplement 1. TiDier Face-to-face and blended cognitive behavioural therapy for MS-related fatigue.**

**Face-to-face Cognitive Behavioural Therapy for MS-related fatigue**

**Why MS-fatigue is a prevalent and burdensome symptom.** The aetiology of MS-related fatigue is likely to be multifactorial. The cognitive behavioural model of MS-related fatigue assumes that disease-specific factors such as (neuro)inflammation trigger fatigue but that cognitive, behavioural and emotional responses can perpetuate fatigue (1). Cognitive behavioural therapy (CBT) for MS-related fatigue aims to decrease fatigue by changing fatigue-related behaviours and beliefs (2, 3). Recent meta-analyses and systematic reviews have shown CBT to be an effective treatment of MS-related fatigue (4-8).

**What** Qualified psychologists were trained in accordance with the TREFAMS-CBT treatment manual (in Dutch: Hans Knoop & Gijs Bleijenberg. Cognitieve gedragstherapie voor chronische vermoeidheid bij MS patiënten – Behandelprotocol. Nijmeegs kenniscentrum chronische vermoeidheid, mei 2011).

**Who provided the therapy** All CBT therapists were state-certified healthcare psychologists who completed a 3-day course on how to provide CBT in accordance with the TREFAMS-CBT protocol. Furthermore, the CBT therapists were supervised every second week by a clinical psychologist with experience in delivering CBT for MS-fatigue. All therapists (24 female and 2 male) were equally qualified and trained in applying CBT for MS-related fatigue, although therapists in the Amsterdam UMC had more experience in treating fatigue in other conditions. The number of therapists per treatment centre varied from 1 to 6. The number of patients treated (face-to-face) by each therapist varied from 1 to 9 (median = 3).

**How, when and how much** Face-to-face CBT consisted of twelve individual, face-to-face, 45-min therapy sessions in 20 weeks

**Where** Sessions took place in the outpatient clinics of 14 participating rehabilitation centres and departments of rehabilitation medicine and medical psychology of (academic) hospitals in The Netherlands (and Belgium)

**Tailoring** CBT for MS-related fatigue is directed at decreasing fatigue by changing fatigue-maintaining cognitions and behaviours. The treatment protocol consisted of ten treatment modules: (1) formulation and attainment of treatment goals; (2) sleep and rest; (3) unhelpful beliefs about MS; (4) unhelpful beliefs about fatigue; (5) focusing on fatigue; (6) physical activity regulation; (7) regulation of social activity; (8) regulation of mental activity; (9) social support; (10) unhelpful beliefs about pain. The CBT is patient-tailored by determining which modules applied to the individual patient on the basis of scores on questionnaires and the clinical judgement of the therapist during the first session. The content of the treatment modules and cut-off scores on questionnaires used for patient-tailoring the modules are described in Table 1.

The subsequent CBT treatment was individualized based on the indicated modules and thus aimed to treat the cognitive-behavioural factors that were thought to maintain or worsen fatigue in an individual patient. After an intake session in which information was given about the cognitive-behavioural model of MS-related fatigue and CBT, patients started with formulating their treatment goals. The following sessions addressed the fatigue-maintaining cognitions and behaviours and were aimed at realizing the set treatment goals. The final therapy sessions focused on integrating the obtained skills into daily life, and on how patients with MS should handle relapses of fatigue.

**Modifications**: Halfway the RCT the Covid-19 pandemic occurred and influenced our original study protocol (9). During the lockdown periods and in accordance with the local measures of the treatment centres, some face-to-face sessions were substituted by video-consultations.

**How well**: Treatment adherence for the face-to-face CBT was defined as attending at least three treatment sessions, including a session at the end of the 20-week period. Therapists registered which modules were indicated and addressed during treatment. In addition, they registered the form (face-to-face, video, phone, email) and duration of each contact.

**Blended Cognitive behavioural therapy for MS-related fatigue (MS Fit)**

**Why** The content of blended CBT is identical to the face-to-face CBT. Since face-to-face CBT draws heavily on treatment capacity and the traveling distance to the treatment centre can be burdensome to severely fatigued patients with MS, we developed a blended CBT, consisting of online treatment modules and a limited number of face-to-face consultations.

**What** The information and assignments in MS Fit were developed by experts on CBT for MS-related fatigue and are based on “Dia-Fit”, an evidence-based blended CBT intervention for severely fatigued patients with Diabetes type 1 (10) and “On the road to recovery”, an evidence-based intervention for cancer-related fatigue in breast cancer survivors (11). The interventions were adapted for MS. Two specific modules about MS, namely unhelpful beliefs about MS, and pain were developed by experts on chronic fatigue and MS. Two patients with MS, who already had received face-to-face CBT for fatigue, and one patient with MS who participates in the Trial Steering Committee on behalf of the Dutch patient organisation MSVN, were asked for usability testing of the portal. Their feedback was used to improve parts of the intervention. MS Fit was provided on a platform of “Minddistrict”.

Qualified psychologists were trained in accordance with the TREFAMS-CBT treatment manual (in Dutch: Hans Knoop & Gijs Bleijenberg. Cognitieve gedragstherapie voor chronische vermoeidheid bij MS patiënten – Behandelprotocol. Nijmeegs kenniscentrum chronische vermoeidheid, mei 2011), and received a 1-day course in applying blended CBT (MS Fit) on the online platform of Minddistrict.

**Who provided the therapy** All therapists in the study provided both face-to-face and blended CBT. See the aforementioned qualifications of the therapists. The number of patients treated (blended) by each therapist varied from 1 to 8 (median = 2).

**How, when and how much** In the blended CBT condition, patients received 2 face-to-face 45-minute consultations with the therapist: the first session (intake) and the last session at week 20. After the first visit, patients received an invitation to MS Fit, which they could start from home. Patients were supported by their therapists with three or four 45-minute video consultations and optional e-mail contact. Therapists stimulated the patients to send an email bi-weekly to inform the therapist on the progress they made in MS Fit, and to ask questions when needed.

**Where** Face-to-face sessions took place in the outpatient clinics of 14 participating rehabilitation centres and departments of rehabilitation medicine and medical psychology of (academic) hospitals in The Netherlands (and Belgium). Patients could follow the treatment and attend the video consultations from home.

**Tailoring** Blended CBT consists of the same treatment modules as face-to-face CBT and is tailored in the same way as described above. Even though the content of the treatment modules is the same, the treatment format is different.

MS Fit consists of 5 standard treatment modules:

1. Formulating treatment goals: introduction, rationale and formulating treatment goals
2. Sleep and rest
3. Fatigue-related cognitions, including focusing on fatigue

- (corresponding with Module 4 and of face-to-face CBT)

1. Activity regulation: physical, mental and social activities

- (corresponding with Module 6, 7 and 8 of face-to-face CBT)

1. Reaching the goals

- (part of Module 1 of face-to-face CBT)

These treatment modules become automatically available to the patients. After formulating treatment goals, module 2 becomes available. After reading the rationale of module 2, module 3 becomes available. Therapists activate module 4 manually, since this treatment module contains two versions and depends on the activity level of the patient (“low active” or “relatively active”). After starting graded activity in module 4, module 5 becomes available.

When indicated, the therapist can activate one or more optional treatment modules as well:

1. Uncertainty about the (consequences of the) illness and appraisal of MS as threatening.
2. Social support
3. Unhelpful thoughts about pain

Treatment modules consist of several “sessions”, in which patients receive information and assignments, and diaries can be used to register activities, bed times, or practice with helpful thoughts. Therapists can view the progress of the patients in the modules and read the diaries. Therapist can provide feedback on the progress made by the patient.

**Modifications**: Halfway the RCT the Covid-19 pandemic occurred and influenced our original study protocol (9). During the lockdowns and in accordance with the local measures of the treatment centres, some face-to-face sessions were substituted by video-consultations.

**How well**: For the blended CBT treatment adherence was defined as attending the first and last treatment sessions, and opening at least the MS Fit modules ‘sleep and rest’ and ‘physical activity regulation’, as an indication that patients actively started the interventions in MS Fit. Therapists registered which modules were indicated and addressed during treatment. In addition, they registered the form (face-to-face, video, phone, email) and duration of each contact. Log data of the online platform provided information about which treatment modules were opened and completed by the patient.

Table 1. CBT modules and assessment tools used for patient tailoring of fatigue treatment

| **Treatment modules** | **Questionnaires and instruments** |
| --- | --- |
| *1. Treatment goals*.  Positive and concrete goals of the fatigue treatment are formulated by each patient. The goals consist of activities they would do when no longer severely fatigued. | All patients |
| *2. Sleep and rest*.  The importance of a regular sleep-wake cycle and a good sleep hygiene are discussed, and patients are helped how to improve this. | Sickness Impact Profile subscale sleep and rest (scores ≥ 60)(12, 13)  Sleep log during one week |
| *3. Uncertainty about the (consequences of the) illness and appraisal of MS* as threatening.  In case of non-accepting cognitions of having MS and extreme fear of the future, the patient is helped to gather realistic information about MS, to develop helping cognitions about MS and the personal future and to develop and maintain a more accepting attitude towards the illness and its consequences. | Impact Event Scale (IES ≥ 20)(14),  Illness Cognition Questionnaire subscale Acceptance (ICQ-acceptance ≤ 12 )(15),  Beck Depression Inventory-PC (>4) (16),  Fear of Disease progression Questionnaire– short form (FoP-Q-SF ≥ 34)(17, 18),  Cognitive behavioural Responses to Symptoms Questionnaire (CBRSQ) (2, 19):   - Resting behaviour > 14.3, - All-or-nothing behaviour > 12.9, - Symptom focusing > 15.5, - Embarrassment > 16.4, - Damage > 20.5, - Fear avoidance > 15.3 |
| *4. Fatigue-related cognitions*.  Sense of control over fatigue symptoms (self-efficacy), fatigue catastrophizing, somatic attributions and other dysfunctional thoughts are assessed(20, 21)(10, 11)(10, 11). Patients formulate more helpful beliefs with respect to fatigue. | modified Self Efficacy Scale for fatigue (≤ 19),  Jacobson-Fatigue Catastrophizing Scale (≥ 16) (22) |
| *5. Focusing on fatigue*.  Information about and consequences of focusing on fatigue will be discussed. Patients will practice with redirecting the focus of attention (away from the fatigue toward activity and other sensations) | Illness Management Questionnaire (≥ 4)(23) |
| *6. Physical activity regulation*.  Depending on the activity pattern patients will learn to spread activities more evenly followed by a gradual increase of regular physical activity (relatively active) or directly start with graded activity (low active patients). After patients have increased their physical activity level they increase other activities in order to reach the goals step by step. | Activity Pattern Interview |
| *7. Regulation of social activity*.  Suggestions are given how to gradually increase social activities and how to handle the problems that are experienced during social interactions (as a consequence of cognitive impairments or intolerance of noise). | Sickness Impact Profile (≥ 100)(13)  SF36 subscale social functioning (≤ 65)(24) |
| *8. Regulation of mental activity*.  Patients are supported with regards to practicing and expanding mental activities such as computer use or reading. They learn how to deal with possible cognitive deficits, such as concentration and memory problems. | CIS20r-concentration subscale (score ≥ 18)(25) |
| *9. Social support*.  Goal of this module is to support emotional independence of others, as far as fatigue is concerned. Unrealistic expectations of others and expressing boundaries are discussed. | The Sonderen Social Support Inventory: subscale discrepancy (score ≥ 50)  subscale negative interactions (score ≥ 14)(26) |
| *10. Unhelpful thoughts about pain*.  Dysfunctional pain cognitions are challenged and more helpful pain cognitions will be installed. | SF36 bodily pain subscale (score ≤ 40)  Pain Catastrophizing Scale (score ≥ 16)(27) |

**References**

1. van Kessel K, Moss-Morris R. Understanding multiple sclerosis fatigue: a synthesis of biological and psychological factors. J Psychosom Res. 2006;61(5):583-5.

2. Knoop H, van Kessel K, Moss-Morris R. Which cognitions and behaviours mediate the positive effect of cognitive behavioural therapy on fatigue in patients with multiple sclerosis? Psychological medicine. 2012;42(1):205-13.

3. van den Akker LE, Beckerman H, Collette EH, Knoop H, Bleijenberg G, Twisk JW, et al. Cognitive behavioural therapy for MS-related fatigue explained: A longitudinal mediation analysis. J Psychosom Res. 2018;106:13-24.

4. Asano M, Finlayson ML. Meta-analysis of three different types of fatigue management interventions for people with multiple sclerosis: exercise, education, and medication. Mult Scler Int. 2014;2014:798285.

5. Harrison AM, Safari R, Mercer T, Picariello F, van der Linden ML, White C, et al. Which exercise and behavioural interventions show most promise for treating fatigue in multiple sclerosis? A network meta-analysis. Mult Scler. 2021;27(11):1657-78.

6. Moss-Morris R, Harrison AM, Safari R, Norton S, van der Linden ML, Picariello F, et al. Which behavioural and exercise interventions targeting fatigue show the most promise in multiple sclerosis? A systematic review with narrative synthesis and meta-analysis. Behav Res Ther. 2019:103464.

7. Phyo AZZ, Demaneuf T, De Livera AM, Jelinek GA, Brown CR, Marck CH, et al. The Efficacy of Psychological Interventions for Managing Fatigue in People With Multiple Sclerosis: A Systematic Review and Meta-Analysis. Front Neurol. 2018;9:149.

8. van den Akker LE, Beckerman H, Collette EH, Eijssen IC, Dekker J, de Groot V. Effectiveness of cognitive behavioral therapy for the treatment of fatigue in patients with multiple sclerosis: A systematic review and meta-analysis. J Psychosom Res. 2016;90:33-42.

9. Houniet-de Gier M, Beckerman H, van Vliet K, Knoop H, de Groot V. Testing non-inferiority of blended versus face-to-face cognitive behavioural therapy for severe fatigue in patients with multiple sclerosis and the effectiveness of blended booster sessions aimed at improving long-term outcome following both therapies: study protocol for two observer-blinded randomized clinical trials. Trials. 2020;21(1):98.

10. Menting J, Tack CJ, van Bon AC, Jansen HJ, van den Bergh JP, Mol M, et al. Web-based cognitive behavioural therapy blended with face-to-face sessions for chronic fatigue in type 1 diabetes: a multicentre randomised controlled trial. Lancet Diabetes Endocrinol. 2017;5(6):448-56.

11. Abrahams HJG, Gielissen MFM, Donders RRT, Goedendorp MM, van der Wouw AJ, Verhagen C, et al. The efficacy of Internet-based cognitive behavioral therapy for severely fatigued survivors of breast cancer compared with care as usual: A randomized controlled trial. Cancer. 2017;123(19):3825-34.

12. Bergner M, Bobbitt RA, Carter WB, Gilson BS. The Sickness Impact Profile: development and final revision of a health status measure. Med Care. 1981;19(8):787-805.

13. Jacobs HM, Luttik A, Touw-Otten FW, de Melker RA. [The sickness impact profile; results of an evaluation study of the Dutch version]. Ned Tijdschr Geneeskd. 1990;134(40):1950-4.

14. van der Ploeg E, Mooren TT, Kleber RJ, van der Velden PG, Brom D. Construct validation of the Dutch version of the impact of event scale. Psychol Assess. 2004;16(1):16-26.

15. Evers AW, Kraaimaat FW, van Lankveld W, Jongen PJ, Jacobs JW, Bijlsma JW. Beyond unfavorable thinking: the illness cognition questionnaire for chronic diseases. J Consult Clin Psychol. 2001;69(6):1026-36.

16. Beck AT, Guth D, Steer RA, Ball R. Screening for major depression disorders in medical inpatients with the Beck Depression Inventory for Primary Care. Behav Res Ther. 1997;35(8):785-91.

17. Herschbach P, Berg P, Dankert A, Duran G, Engst-Hastreiter U, Waadt S, et al. Fear of progression in chronic diseases: psychometric properties of the Fear of Progression Questionnaire. J Psychosom Res. 2005;58(6):505-11.

18. Kwakkenbos L, van den Hoogen FH, Custers J, Prins J, Vonk MC, van Lankveld WG, et al. Validity of the Fear of Progression Questionnaire-Short Form in patients with systemic sclerosis. Arthritis Care Res (Hoboken). 2012;64(6):930-4.

19. Dennison L, Moss-Morris R, Silber E, Galea I, Chalder T. Cognitive and behavioural correlates of different domains of psychological adjustment in early-stage multiple sclerosis. J Psychosom Res. 2010;69(4):353-61.

20. Skerrett TN, Moss-Morris R. Fatigue and social impairment in multiple sclerosis: the role of patients' cognitive and behavioral responses to their symptoms. J Psychosom Res. 2006;61(5):587-93.

21. Strober LB, Arnett PA. An examination of four models predicting fatigue in multiple sclerosis. Arch Clin Neuropsychol. 2005;20(5):631-46.

22. Jacobsen PB, Andrykowski MA, Thors CL. Relationship of catastrophizing to fatigue among women receiving treatment for breast cancer. J Consult Clin Psychol. 2004;72(2):355-61.

23. Ray C, Weir W, Stewart D, Miller P, Hyde G. Ways of coping with chronic fatigue syndrome: development of an illness management questionnaire. Soc Sci Med. 1993;37(3):385-91.

24. Aaronson NK, Muller M, Cohen PD, Essink-Bot ML, Fekkes M, Sanderman R, et al. Translation, validation, and norming of the Dutch language version of the SF-36 Health Survey in community and chronic disease populations. J Clin Epidemiol. 1998;51(11):1055-68.

25. Vercoulen JH, Bazelmans E, Swanink CM, Fennis JF, Galama JM, Jongen PJ, et al. Physical activity in chronic fatigue syndrome: assessment and its role in fatigue. J Psychiatr Res. 1997;31(6):661-73.

26. van Sonderen E. Sociale Steun Lijst–Interacties (SSL-I) en Sociale Steun Lijst-Discrepanties (SSL-D). 1993.

27. Sullivan MJ, Bishop SR, Pivik J. The pain catastrophizing scale: development and validation. Psychological assessment. 1995;7(4):524.
